# Initiation of a lightning search using the lightning and airglow camera onboard the Venus orbiter Akatsuki

**DOI:** 10.1186/s40623-018-0836-2

**Published:** 2018-05-25

**Authors:** Yukihiro Takahashi, Mitsuteru Sato, Masataka Imai, Ralph Lorenz, Yoav Yair, Karen Aplin, Georg Fischer, Masato Nakamura, Nobuaki Ishii, Takumi Abe, Takehiko Satoh, Takeshi Imamura, Chikako Hirose, Makoto Suzuki, George L. Hashimoto, Naru Hirata, Atsushi Yamazaki, Takao M. Sato, Manabu Yamada, Shin-ya Murakami, Yukio Yamamoto, Tetsuya Fukuhara, Kazunori Ogohara, Hiroki Ando, Ko-ichiro Sugiyama, Hiroki Kashimura, Shoko Ohtsuki

**Affiliations:** 1 Graduate School of Science, Hokkaido University, Kita 10 Nishi 8, Kita-ku, Sapporo, Hokkaido 060-0810 Japan; 2 Johns Hopkins University Applied Physics Lab, 11100 Johns Hopkins Road, Laurel, MD 20723 USA; 3 School of Sustainability, Interdisciplinary Center Herzliya (IDC), P. O. Box 167, 46150 Herzliya, Israel; 4 Physics Teaching Laboratories, Oxford University, Denys Wilkinson Building, Keble Rd, Oxford, OX1 3RH UK; 5 Space Research Institute, Austrian Academy of Sciences, Schmiedlstr. 6, 8042 Graz, Austria; 6 Institute of Space and Astronautical Science, Japan Aerospace Exploration Agency, 3-1-1 Yoshinodai, Chuo-ku, Sagamihara, Kanagawa 252-5210 Japan; 7 Department of Space and Astronautical Science, School of Physical Sciences, SOKENDAI, 3-1-1 Yoshinodai, Chuo-ku, Sagamihara, Kanagawa 252-5210 Japan; 8 Graduate School of Frontier Sciences, The University of Tokyo, Kiban-tou 4H7, 5-1-5 Kashiwanoha, Kashiwa, Chiba 277-8561 Japan; 9 Department of Earth Science, Okayama University, 3-1-1 Tsushimanaka, Kita, Okayama, 700-8530 Japan; 10 School of Computer Science and Engineering, The University of Aizu, 90 Kami-Iawase, Tsuruga, Ikki-machi, Aizu-Wakamatsu, Fukushima 965-8580 Japan; 11 Department of Earth and Planetary Science, Graduate School of Science, The University of Tokyo, Hongo 7-3-1, Bunkyo-ku, Tokyo, 113-0033 Japan; 12 Planetary Exploration Research Center, Chiba Institute of Technology, 2-17-1 Tsudanuma, Narashino, Chiba 275-0016 Japan; 13 Department of Physics, Rikkyo University, 3-34-1 Nishi-Ikebukuro, Toshima-ku, Tokyo, 171-8501 Japan; 14 School of Engineering, University of Shiga Prefecture, 2500 Hassaka-cho, Hikone, Shiga 522-8533 Japan; 15 Faculty of Science, Kyoto Sangyo University, Motoyama, Kamigamo, Kita-ku, Kyoto, Kyoto 603-8555 Japan; 16 Department of Information Engineering, National Institute of Technology, Matsue College, 14-4 Nishi-Ikuma, Matsue, Shimane 690-8518 Japan; 17 Japan Agency for Marine-Earth Science and Technology, 3173-25 Showa-machi, Kanazawa-ku, Yokohama, Kanagawa 236-0001 Japan; 18 School of Commerce, Senshu University, 2-1-1 Higashimita, Tama-ku, Kawasaki, Kanagawa 214-8580 Japan

**Keywords:** Venus, Lightning, Flash, Akatsuki, Lightning and airglow camera

## Abstract

The existence of lightning discharges in the Venus atmosphere has been controversial for more than 30 years, with many positive and negative reports published. The lightning and airglow camera (LAC) onboard the Venus orbiter, Akatsuki, was designed to observe the light curve of possible flashes at a sufficiently high sampling rate to discriminate lightning from other sources and can thereby perform a more definitive search for optical emissions. Akatsuki arrived at Venus during December 2016, 5 years following its launch. The initial operations of LAC through November 2016 have included a progressive increase in the high voltage applied to the avalanche photodiode detector. LAC began lightning survey observations in December 2016. It was confirmed that the operational high voltage was achieved and that the triggering system functions correctly. LAC lightning search observations are planned to continue for several years.
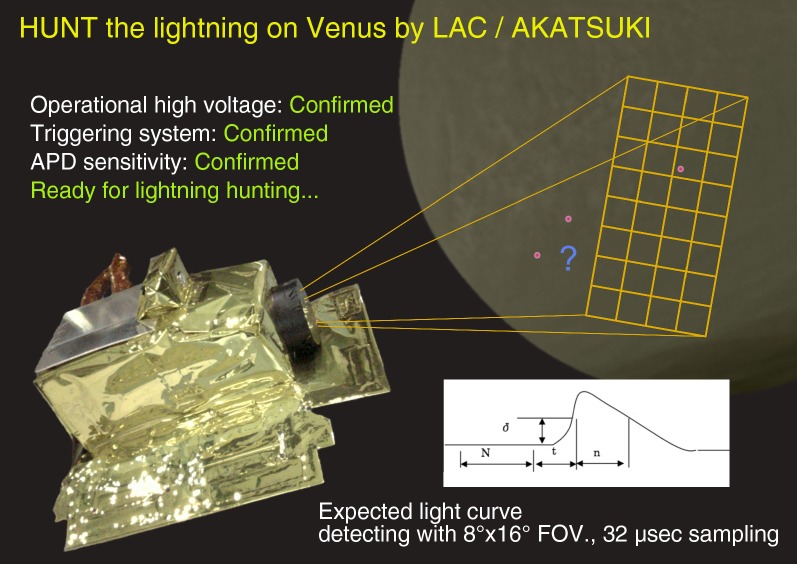

## Introduction

Observations detecting potential lightning flashes on Venus have been attempted for more than 30 years, with both optical and radio wave sensors, and by spacecraft and ground-based telescopes, as reviewed by Takahashi et al. (2008). However, the scientific community has not yet reached a consensus on the existence of lightning on the planet. Among the principal reasons for the ongoing controversy may be the challenges in discriminating natural lightning signatures from transient noise caused by other sources. Receivers onboard spacecraft in the very low frequency (VLF) and high frequency (HF) ranges have recorded transient signals possibly emitted from lightning channels (e.g., Russell 1991; Gurnett et al. 1991, 2001). However, such waves could also have been generated by other sources, such as from spacecraft equipment, discharge from the spacecraft body to the space environment, or space plasma instabilities. Borucki et al. (1981, 1991) investigated data acquired by the Star Tracker on the Pioneer Venus Orbiter, which was designed to detect star light to determine the attitude of the spacecraft but was also expected to be sensitive to optical lightning flashes. However, this sensor also records pulses caused by cosmic rays incident on the detector, and given the sensor's electronics cannot be distinguished from a lightning flash. The authors attempted to identify a statistically significant increase in such pulses during night side disk viewing, but found no meaningful indication of lightning. Gurnett et al. (1991) also examined the statistical distribution of pulse height measured using an HF receiver during the Venus flyby of the Galileo spacecraft and reported nine possible lightning pulses with pulse heights greater than 4 sigma of background. On the other hand, Gurnett et al. (2001) during two Venus flybys of the Cassini spacecraft found no evidence for sferics induced by lightning discharge (whereas the same instrumentation easily detected lightning emissions during a later Earth flyby). Hansell et al. (1995) detected several candidate lightning flashes using a 1.5-m ground-based telescope. The imaging rate of the charge-coupled device (CCD) camera was 18.8 frames/s, which is not sufficiently rapid to take more than one frame for one lightning flash. Therefore, there remains some ambiguity in deciding whether the recorded light spot originated as lightning as cosmic rays or other unknown electrical noise could cause similar apparent features.

Krasnopolsky (1983a, b) observed random flashing during a single 70-s period of observations of the Venera 9 visible airglow spectrometer; the corresponding Venera 10 instrument recorded no such flashes. If the Venera 9 signals indeed corresponded to lightning, they occurred in a region of 5 × 10^4^ km^2^ with a flashing rate of 0.002 km^−2^ s^−1^, a mean flash duration of 0.25 s, and a flash energy of 2 × 10^7^ J in the visible range. Krasnopolsky (2006) reported detection of nitric oxide (NO) in Venus clouds and argued that lightning was the only plausible source of NO in the lower atmosphere (although he noted some uncertainty in relevant reaction rates and did not quantify meteoric production).

Among the best means to distinguish a lightning signature from possible noise is to inspect the light curve (or ‘waveform’) of a lightning flash sampled at a cadence sufficiently high compared to the expected duration of the flash. Such a waveform, whose shape is determined in part by the distribution of light propagation paths through the cloud, can be diagnostic of the depth at which the discharge occurs. On the other hand, a cosmic ray strike on the detector has an instantaneous rise and a decay constant was determined only by the instrumentation electronics.

## Instrumentation and observation strategy

**Fig. 1:**
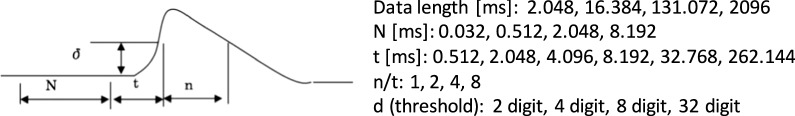
Schematic drawing of triggering logic

LAC is the first fast-sampling optical sensor designed to measure the light curve of lightning flashes on a planet other than the Earth. To achieve an adequate sampling rate, we sacrificed spatial resolution such that the avalanche photodiode (APD) format was only 4 × 8 pixels (totaling 32 pixels). Though the basic specifications of the instrument are the same as those listed in Takahashi et al. (2008), the sampling rate was modified from 50 to 31.25 kHz for the flight model. Considering that the duration of optical lightning strokes measured using photometers of the Japanese Experiment Module (JEM)/Global Lightning and sprIte Measurements (GLIMS) instrument on the International Space Station is on the order of one millisecond, the LAC sampling interval of 30 μs is considered adequate. The field of view is 8 × 16°, corresponding to 700 km × 1400 km on the surface of Venus at distance of 5000 km, which is approximately the average of the closest range for all of the observations. The data recording and triggering logic were designed to accommodate various types of flash waveforms, given the unknown parameters of Venus lightning (Fig. 1). The triggering logic works using every pixel, and data were recorded only for the pixel triggered first. The recorded data length can be chosen from 2.048 ms to 2.096 s. The time duration to define the background level (*N* in Fig. 1) was from 0.02 to 8.192 ms. The time gap between the last of the background sampling and the triggering timing (*t*) was from 0.512 to 32.768 ms. The threshold level was defined at 4 levels, while the sensitivity was also controlled by the high voltage to the APD. The necessary consecutive duration over the threshold for triggering (*n*) was defined as *n*/*t* from 1 to 8. With this highly flexible combination of parameters, it is possible to trigger and store data for various types of optical phenomena, including not only typical cloud-to-ground or intra-cloud lightning discharges but also transient luminous events (TLEs) in the middle and upper atmosphere, termed sprites, elves, blue and gigantic jets, etc. The possibility of the occurrence of TLEs on Venus was discussed by Yair et al. (2009) and Pérez-Invernón et al. (2016). For the first trial, we chose a parameter set consisting of data length = 16.384 ms, *N* = 0.512 ms, *t* = 0.512 ms, *n*/*t* = 2, and a threshold = 2 digits, which is optimized for normal lightning detection. Absolute calibration for the sensitivity of an individual pixel was conducted using a 2-m integrating sphere at the National Institute for Polar Research, Japan. The results showed a variation in relative sensitivity of approximately ± 50% from average. We use a typical value for the discussion here.

We selected the oxygen OI-777-nm line for lightning detection, which was expected to be the most prominent emission in a CO_2_-dominant atmosphere based on laboratory experiments (Borucki et al. 1985). The bandwidth (FWHM) and center wavelength of the LAC OI-777-nm filter were 9.0 and 780.6 nm, respectively. If the spacecraft is 5500 km above Venus surface, the threshold of triggering is approximately 1/20 of the average luminosity of an Earth lightning flash and the instantaneous field of view (FOV) is 1/500 of the whole globe. Here, we assume that approximately 40% of the total optical emission is from the OI-777-nm line according to the photographic spectra in Borucki et al. (1985). However, Krasnopolsky (2006) noted that OI 777 nm is approximately 3% of the total light in the more sensitive photoelectric spectra of Borucki et al. (1996), where the bright continuum dominates: In this case, our detection threshold would be higher, comparable to the average lightning on Earth.

## Orbital condition for observation and setup of high voltage for the APD

Akatsuki was successfully inserted into orbit surrounding Venus during December 2016, but with a period of ~ 10 days, approximately ~ 8 times longer than the originally intended orbit. LAC can be powered only inside the umbra of Venus because the strong sunlight can cause damage to its detector. Therefore, the frequency of observation opportunities was reduced to 1/8 of that of the original plan. Adding to this decrease in observational frequency, the elongated orbit resulted in a more rapid motion of spacecraft near periapsis where LAC can observe the night side disk at a close distance, meaning that the duration of observation for one revolution was shorter than that of the original. Indeed, most of the observational periods were approximately 20–30 min, while the original plan anticipated sessions a few times longer. Thus, the total observational period per year was reduced to less than 10% of that of the original plan because of the change in the orbital parameters. On the other hand, the successful insertion into orbit around Venus, 5 years later than the original plan, allowed for observations that would otherwise be completely impossible and promises with time to establish significant constraints on lightning activity on Venus.

**Fig. 2:**
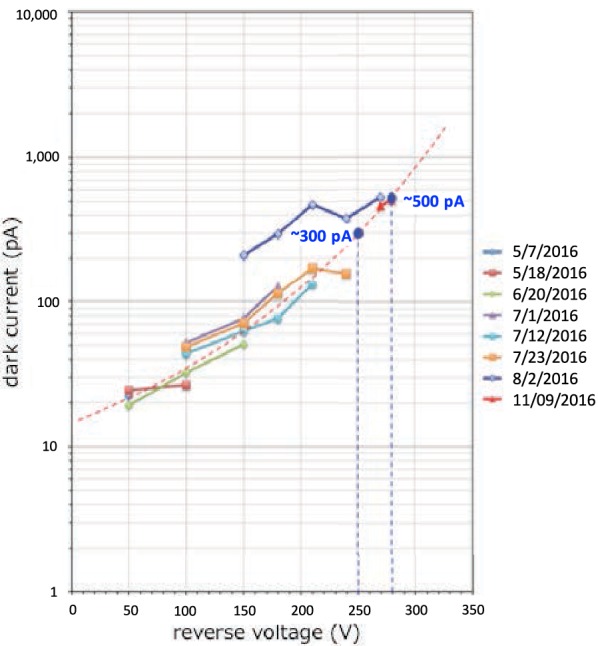
Relationship between the reverse voltage applied to APD and the dark currents measured during 8 days from May 7 to November 9, 2016

The most important work in the start-up operation of LAC was the increase in high-voltage power supply to the APD. Because the high-voltage power supply unit had not been turned on since the launch in 2010, careful operation was required and the high voltage was increased only gradually, over 10 steps. As previously mentioned, the operation was conducted only during the short duration of the umbra of Venus; thus, each increment was performed during a different 10-day orbit. Figure 2 shows the relationship between the reverse voltage applied to the APD and the dark current of output for the operations from May 7 to November 9, 2016. The dark current was approximately on the expected curve (red dotted line), while the deviation from the line was considered to be caused mainly by temperature fluctuations of the APD.

We chose 300 V as the applied voltage to the APD for regular lightning observations for the time being. Considering the level of dark current, the APD seems to be working properly without serious problems. At this voltage, the sensitivity of LAC is expected to be sufficient to detect an optical lightning flash with an intensity of 0.05 to 0.5 of the average Earth flash at a distance of 5500 km, depending on its spectral distribution. At the longer range of our observations of 12,000 km, the threshold could be as high as 2× that of the average Earth flash.

## Preliminary observations

The lightning search at a nominal high voltage of 300 V was attempted 8 times in the lightning observation mode of LAC with each duration of  ~ 10 to 30 min on December 1, 2016. The distance to the Venus surface was in the range of ~ 7500 to 12,300 km, meaning the averaged instant coverage was approximately 1/500 of the whole globe and the threshold of the triggering changed by ~ 1.6 times in this case. There were some triggered events every pass. However, all showed a steep increase within two sampling intervals (< 63 us, meaning they were caused by cosmic ray impact. Thus far, no signature of lightning has been identified. It was confirmed that all 32 pixels for lightning measurement detected cosmic ray events and the triggering logic for all pixels works properly. In addition, if some pixels were exposed to the limb of Venus illuminated by scattered sunlight, they recorded a reasonable light curve. Based on this and the increase in dark currents as the high voltage was ramped up, we consider LAC works overall as expected.

**Fig. 3:**
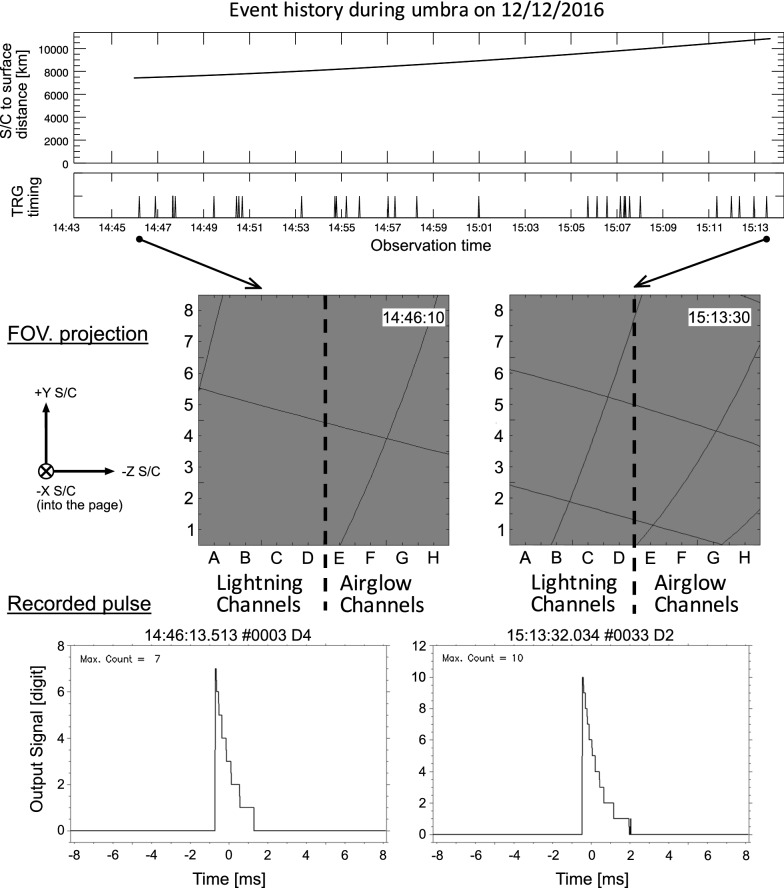
An example of one pass observation. The top and second panels show the distance from Venus surface and the occurrence of triggered events for cosmic rays (CR) and lightning (LIG). The third and fourth panels express LAC’s FOV and examples of time variation during a cosmic ray event

Figure 3 shows an example of the quick look data from one operation. The upper panel indicates the distance from the Venus surface and the second the time series of triggered pulses. At each pulse time, we checked the projection of LAC’s FOV onto Venus. (The lightning observing area is rows A–D.) The bottom plots are examples of waveforms caused by cosmic rays.

The heights of the cosmic ray pulses are consistent with the triggering level set in the lightning observation mode, implying a normal condition of triggering logic and hardware. All the curves show the same decay period of 1.8 ms, which was expected from the design of the electronic circuit and temperature of the APD. Considering these conditions, the health and performance of LAC are quite sufficient to capture a lightning flash if it were to occur inside the FOV at brightness greater than the threshold of the instrument as designed.

## Future work

Given that cosmic ray events are triggered at our chosen threshold values and recorded properly, and the dim light near the limb caused triggering by the designed logic and provided saturated signals, the optical and electronic performance of LAC—including its sensitivity—appears to be normal, even after prolonged flight in space. However, it would be desirable to measure the absolute sensitivity from on-orbit observational data on Venus in the future. Among the ideas is to measure the Earth which is the only celestial target sufficiently bright to detect in the lightning observation mode of LAC. Another possibility would be to observe the limb together with the ultraviolet imager (UVI) onboard Akatsuki and compare it to the UVI image whose sensitivity can be calculated from star images.

We expect to continue the lightning search operations for several years. To increase the possibility of capturing events, we plan to concentrate the dusk area of the night side disk where the thunderstorm activity may be higher than at other local times according to experience on Earth. Indeed, 5 of the 8 possible lightning events reported by Hansell et al. (1995) and Krasnopolsky (1983a, b) were found in the dusk region on Venus. We also plan to focus our search for optical transients near candidate volcanos: Volcanic plumes might result in convective activity in clouds, or (as on Earth) there may be discharge within the plume itself. Hashimoto and Imamura (2001) discuss the possibility of volcanic detection using an infrared camera onboard Akatsuki. Russell (1991) reviewed possible lightning sources including active volcanos.

## Conclusion

Initial operations of LAC including the operational level of high voltage applied to the APD have been successfully completed. The threshold of optical detection is 0.05–2 times that of the averaged intensity of Earth’s lightning. The performance of LAC seems nominal, as evidenced by the detection and correct recording of cosmic rays and dim luminosity near the limb. Though the duty cycle of the lightning observation time in the present orbit is > 10 times less than that in the originally planned orbit of Akatsuki, continued operation over the coming years will provide a statistically robust dataset.

## References

[CR1] Borucki WJ, Dyer JW, Thomas GZ, Jordan JC, Comstock DA (1981). Optical search for lightning on Venus. Geophys Res Lett.

[CR2] Borucki WJ, Mc Kenze RL, McKay CP, Duong ND, Boac DS (1985). Spectra of simulated lightning on Venus, Jupiter, and Titan. Icarus.

[CR3] Borucki WJ, Dyer JW, Phillips JR, Phan P (1991). Pioneer Venus orbiter search for Venusian lightning. J Geophys Res.

[CR4] Borucki WJ, McKay CP, Jebens D, Lakkaraju HS, Vanajakshi CT (1996). Spectral irradiance measurements of simulated lightning in planetary atmospheres. Icarus.

[CR5] Gurnett DA, Kurith WS, Roux A, Gendrin R, Kennel CF, Bolton SJ (1991). Lightning and plasma wave observations from the Galileo flyby of Venus. Science.

[CR6] Gurnett DA, Zarka P, Manning R, Kurth WS, Hospodarsky GB, Averkamp TF (2001). Non-detection at Venus of high-frequency radio signals characteristic of terrestrial lightning. Nature.

[CR7] Hansell SA, Wells WK, Hunten DM (1995). Optical detection of lightning on Venus. Icarus.

[CR8] Hashimoto G, Imamura T (2001). Elucidating the rate of volcanism on Venus: detection of lava eruptions using near-infrared observations. Icarus.

[CR9] Krasnopolsky VA, Hunten DM (1983). Venus spectroscopy in the 3000–8000. A region by Veneras 9 and 10. Venus.

[CR10] Krasnopolsky VA (1983). Lightnings and nitric oxide on Venus. Planet Space Sci.

[CR11] Krasnopolsky VA (2006). A sensitive search for nitric oxide in the lower atmospheres of Venus and Mars: detection on Venus and upper limit for Mars. Icarus.

[CR12] Pérez-Invernón FJ, Luque A, Gordillo-Vázquez FJ (2016). Mesospheric optical signatures of possible lightning on Venus. J Geophys Res Space Phys.

[CR13] Russell CT (1991). Venus lightning. Space Sci Rev.

[CR14] Takahashi T, Yoshida J, Yair Y, Imamura T, Nakamura M (2008). Lightning detection by LAC onboard the Japanese Venus climate orbiter, planet-C. Space Sci Rev.

[CR15] Yair Y, Takahashi Y, Yaniv R, Ebert U, Goto Y (2009). A study of the possibility of sprites in the atmospheres of other planets. J Geophys Res.

